# TRIB1 regulates tumor growth via controlling tumor-associated macrophage phenotypes and is associated with breast cancer survival and treatment response

**DOI:** 10.7150/thno.72192

**Published:** 2022-04-24

**Authors:** Taewoo Kim, Jessica Johnston, Sonia Castillo-Lluva, Francisco J. Cimas, Stephen Hamby, The Cardiogenics Consortium*, Santiago Gonzalez-Moreno, Pedro Villarejo-Campos, Alison H Goodall, Guillermo Velasco, Alberto Ocana, Munitta Muthana, Endre Kiss-Toth

**Affiliations:** 1 Department of Infection, Immunity and Cardiovascular Diseases, University of Sheffield Medical School, Sheffield, S10 2RX, UK.; 2 Department of Biochemistry and Molecular Biology, Complutense University and Instituto de Investigación Sanitaria Clínico San Carlos (IdISSC), 28040, Madrid, Spain.; 3 Hospital Clínico San Carlos (HCSC), Instituto de Investigación Sanitaria Clínico San Carlos (IdISSC), Madrid and Universidad de Castilla La Mancha (UCLM), Albacete, Spain.; 4 Department of Cardiovascular Sciences, Glenfield Hospital, University of Leicester and Leicester NIHR Biomedical Research Centre, Leicester, UK.; 5 MD Anderson Cancer Center Madrid, Madrid, Spain.; 6 Hospital Universitario Fundación Jiménez Díaz, Madrid, Spain.; 7 Department of Oncology and Metabolism, University of Sheffield Medical School, Sheffield, S10 2RX, UK.; 8 Biological Research Centre of the Hungarian Academy of Sciences, Temesvari krt. 62, Szeged, 6726, Hungary.

**Keywords:** Breast cancer, tumor associated macrophages, tribbles, TRIB1, response to chemotherapy, interleukin 15

## Abstract

Molecular mechanisms that regulate tumor-associated macrophage (TAM) phenotype and function are incompletely understood. The pseudokinase TRIB1 has been reported as a regulator of macrophage phenotypes, both in mouse and human systems.

**Methods:** Bioinformatic analysis was used to investigate the link between *TRIB1* expression in breast cancer and therapeutic response to chemotherapy. *In vivo* models of breast cancer included immune-competent mice to characterize the consequences of altered (reduced or elevated) myeloid *Trib1* expression on tumor growth and composition of stromal immune cell populations.

**Results:** TRIB1 was highly expressed by TAMs in breast cancer and high *TRIB1* expression correlated with response to chemotherapy and patient survival. Both overexpression and knockout of myeloid *Trib1* promote mouse breast tumor growth, albeit through different molecular mechanisms. Myeloid *Trib1* deficiency led to an early acceleration of tumor growth, paired with a selective reduction in perivascular macrophage numbers *in vivo* and enhanced oncogenic cytokine expression *in vitro*. In contrast, elevated levels of *Trib1* in myeloid cells led to an increased late-stage mammary tumor volume, coupled with a reduction of NOS2 expressing macrophages and an overall reduction of macrophages in hypoxic tumor regions. In addition, we show that myeloid *Trib1* is a previously unknown, negative regulator of the anti-tumor cytokine IL-15, and that increased myeloid *Trib1* expression leads to reduced IL-15 levels in mammary tumors, with a consequent reduction in the number of T-cells that are key to anti-tumor immune responses.

**Conclusions:** Together, these results define a key role for TRIB1 in chemotherapy responses for human breast cancer and provide a mechanistic understanding for the importance of the control of myeloid TRIB1 expression in the development of this disease.

## Introduction

Breast cancer (BC), the leading cause of cancer death in females 1, is initiated by the formation of a tumor niche where cancer-initiating cells or breast cancer stem cells recruit healthy, non-transformed cells 2, 3. These cells are re-educated by signals released from cancer cells to promote the expression of oncogenic cytokines and growth factors 4. Tumor-associated macrophages (TAMs) are one of the most abundant cell types that can comprise up to 50% of the tumor microenvironment (TME) and facilitate tumor initiation and development 5. A number of published studies reported high infiltration of TAMs in breast cancer, correlating with poor prognosis and clinical outcomes 6-9. Triple-negative breast cancer (TNBC) tumors have been shown to have a higher number of CD68^+^ macrophages compared to other subgroups 10.

The plasticity of infiltrated TAMs is influenced by environmental signals and can be functionally classified into M1 (pro-inflammatory) and M2 (anti-inflammatory) cells, as two extremes 9. Though TAMs are able to express markers of either polarization phenotype, pro-inflammatory (M1-like) macrophages are generally observed upon entering to the tumor site 11, but these macrophages, stimulated by the type 1 T helper cell (T_h_1) cytokines are known to exhibit anti-tumor capacity by generating anti-tumor cytokines (such as TNF, IL-2, and IL-12) and reactive nitrogen and oxygen intermediates 9, 12, 13. Most TAMs are polarized to have a M2-like phenotype after infiltration and produce anti-inflammatory cytokines (such as IL-4) and growth factors to inhibit immune response and promote proliferation 14. These M2-like TAMs have been associated with unfavorable clinical outcomes and patient survival 15.

Hypoxia promotes the dephosphorylation of chemoattractant receptors and inhibits migrating stimulating factors that trap TAMs in the hypoxic area and is associated with aggressive breast tumor phenotypes 14, 16. The entrapped, hypoxic TAMs facilitate tumor vascularization and immune suppression by expressing angiogenic molecules and immunosuppressive factors, respectively 16, 17.

The pseudokinase Tribbles-1 (TRIB1) is highly expressed in the macrophage lineage and has been shown to regulate macrophage polarization 18-20 and *Trib1*-deficient mice were shown to lack anti-inflammatory macrophages 21-23. Previous studies reported *TRIB1* as an oncogene 24-26, including in prostate and colon cancer but its mechanistic contribution to tumorigenesis is yet to be fully understood.

Using Bayesian network inference modelling, TRIB1 expression was shown to be correlated with the levels of NF- κB and IL-8 in breast cancer, and was also considered as a potential biomarker for clinical outcomes 27. However, unlike recent papers reporting an oncogenic role of TRIB1 in prostate cancer via regulating macrophage infiltration and inducing M2-like polarization 28, to date no study has examined the TAM-specific tumoral capacity dependence on *Trib1* and how this may influence tumor development. Therefore, we analyzed the impact of TRIB1 on the survival of BC patients and showed that mutations or reduced expression of this gene are associated with a poor clinical prognosis, response to therapy and survival. We also found that TRIB1 highly expressed by TAMs, both in human BC and murine model. Prompting us to study mammary tumor development in mice where levels of myeloid*-Trib1* (*mTrib1*) have been genetically altered. Based on our analyses, we demonstrate that both overexpression and knockout of *mTrib1* promote tumor growth, albeit through distinct molecular mechanisms and at different stages of tumor growth, providing a novel mechanistic insight into the functional importance of TAM phenotypes.

## Methods

### Study approval

All animal studies were approved and conducted in accordance with the University of Sheffield code of ethics and Home Office regulations (project license No. PPL70/8670). Human monocyte-derived macrophages (MDMs) isolated from healthy participants were obtained with signed participant-informed consent and approval from the University of Sheffield Research Ethics Committee (project license No. SMBRER310) and in accordance with the Declaration of Helsinki. All participants gave written informed consent. The TRIB1-CD68 co-staining was studied by immunofluorescence in breast tumor samples from patients according to the Declaration of Helsinki. Studies were performed after approval of The Bioethics Committee at The MD Anderson Cancer Center Madrid (MD21/004). Written informed consent was obtained from each patient.

### Bioinformatics analysis of human BC transcriptomes

In order to establish a correlation between mutations and patient clinical outcome, Genotype-2-Outcome (http://www.g-2-o.com) algorithms were used, as previously described 29. This approach calculates the prognosis conferred by a specific transcriptomic signature linked with a mutation and patient survival. Briefly, 763 breast cancer patients with NGS data publicly available from TCGA were collected and classified in terms of TRIB1 mutation status (functionally annotated using SNPeff v3.5 30 and including just somatic mutations), considering only the ones labelled as 'KEEP' by the MutTect judgment algorithm and present in at least four reads with a minimum of 20-fold read coverage. Two cohorts, the wild type and the mutant, are defined and the transcriptomic signature by univariate receiver operating characteristic (ROC) analysis performed separately for each gene and significantly altered genes are selected by their area under the curve value (calculated by the ROCR package), and the associated P value obtained from the ROC analysis, considering the null hypothesis where AUC value equals to 0.5 and only genes passing both AUC and P value thresholds are considered significant. The median expression values for different transcripts are used as a cut-off to discriminate “high” and “low” expression cohorts, which are compared using a Cox survival analysis (proportional hazards) and graphics are drawn using ggplot2 package running in R Studio Version 1.2.5033. To calculate prognosis under treatment, gene expression and therapy response are compared using receiver operating characteristics and Mann-Whitney test t or ROC test in the R statistical environment (www.r-project.org) using Bioconductor libraries. Statistical significance was set at p < 0.05 in both cases.

### Microarray analysis

The Cardiogenic Transcriptomic Study 31-33 was analyzed as described in 19. In brief, top and bottom quartiles of TRIB1 expressing monocytes (n = 758) and macrophages (n = 596) were compared and obtained the TRIB1 co-regulated, differentially expressed genes using FDR adjusted p-values of < 0.01, cut-off log-2 fold changes of > 0.071 (upregulated) and > -0.071 (down-regulated). The gene list was further analyzed with QuSage 34-36 to identify the pathways enriched in the TRIB1 co-expressed gene sets.

### Mice

All mice were bred on a *C57BL/6* genetic background under the University of Sheffield code of ethics, and Home Office regulations in the University of Sheffield Biological Service Unit. Trib1 fl/fl × Lyz2Cre (*Trib1^mKO^*), ROSA26.Trib1Tg × Lyz2Cre (*Trib1^mTg^*) and their corresponding WT controls have recently been described 19.

### Tumor models

The mouse Basal-B BC cell line, E0771 37 (obtained from Dr Jessalyin Ubellacker (University of Harvard, USA) was cultured in DMEM medium (Gibco) containing 10% (v/v) low endotoxin heat-inactivated fetal bovine serum (Biowest), and 1% L-glutamine (Lonza). Eight-week-old female *Trib1^mKO^* and *Trib1^mTg^* mice were inoculated with 3 × 105 E0771 cells into the right nipple via intra-ductal injection. Once the tumors formed, the size was measured every 2 days with calipers until it reached 15 mm in diameter. Data was accumulated from >5 independent experiments, each containing several litter-mates, including both wild type and m*Trib1*-altered mice. Samples were harvested at the end of each experiment and were processed/analyzed for either as a batch (FACS) or together (immunofluorescence, qRT-PCR), as appropriate.

### Cancer cell culture and conditioned medium production

MDA-MB-231, BT474, SKBR3 and MCF7 cell lines were cultured in RPMI-1640 (Gibco) with 10% (v/v) low endotoxin heat-inactivated fetal bovine serum (LE-FBS) (Biowest), 1% (v/v) streptomycin/penicillin (Gibco), 1% L-glutamine (Lonza). All cells were obtained from Dr. Penelope Ottowell and Dr. Munitta Muthana (University of Sheffield, UK) and subsequently maintained in our laboratory. Routinely testing for mycoplasma contamination demonstrated they were consistently negative. To obtain MDA-MB-231 conditioned medium (CM), cells were cultured in T75 flasks for 48 hours at 37 °C in a 5% CO2 and the supernatant centrifuged at 600 × g for 5 minutes to remove cells and cellular debris (but not extracellular vesicles released by these cells).

### Isolation of human blood monocytes

Whole blood was collected in 3.8% trisodium citrate (Sigma) and used immediately to isolate cells. In 15 ml of Ficoll-Paque PLUS (GE Healthcare), 30 ml of blood was gently layered and centrifuged at 900 × g for 20 minutes at room temperature (RT) to separate peripheral blood mononuclear cells (PBMCs) from plasma. PBMCs were recruited in PBS-EDTA (Thermo Fischer) solution (PBSE) and centrifuged at 400 × g for 5 minutes at RT. After red blood cell lysis with 10 ml of RBC lysis buffer (155 mM NH4Cl, 10 mM KHCO3, 0.1M EDTA in H2O) for 5 minutes at RT, 40 ml of PBSE was added and centrifuged at 1500 rpm or 400 × g for 5 minutes. Cells were counted using a hemocytometer (Hawksley) and resuspended in 90 μl 4 °C MACS buffer (0.5% [w/v] bovine serum albumin [BSA, Sigma] - PBSE) and 10 μl CD14+ microbeads (Miltenyi Biotec) per 10^7^ cells for 15 minutes at 4 °C. 2 ml of MACS buffer was added and centrifuged at 260 × g for 5 minutes. CD14+ monocytes were isolated with LS column (Miltenyi Biotec) and MidiMACSTM Separator (Miltenyi Biotec) for differentiation.

### TRIB1 siRNA transfection

Viromer Green (Lipocalyx) was used to transfect TRIB1 siRNA (ON-TARGET plus siRNA, Dharmacon) and Non-Targeting Control siRNA (ON-TARGET plus siRNA, Dharmacon) in order to knockdown TRIB1 level in humans MDMs according to the manufacturer's instructions.

### Monocyte-derived macrophages differentiation and stimulation

Isolated monocytes were incubated in fresh medium (RPMI-1640 (Gibco) 10% (v/v) LE-FBS (Biowest), 1% (v/v) streptomycin/penicillin (Gibco), 1% L-glutamine (Lonza)) with 100 ng/ml recombinant human (rh) macrophage-colony stimulating factor (M-CSF) (Peprotech) for 7 days at 37 °C, at 5% CO2 to facilitate differentiation of monocytes to macrophages. MDMs were washed with PBS and polarized by incubating with 20 ng/ml IFN-γ (Peprotech) and 100 ng/ml E. coli lipopolysaccharide (Serotype R515 TLRgradeTM, Enzo Life Sciences), 20 ng/ml IL-4 (Peprotech), 20 ng/ml IL-10 (Peprotech), and CM for 24 hours at 37 °C, at 5% CO2.

### Isolation of BMDMs

The femur and tibias of mice were collected, and tissues were gently removed from the bones. Bone marrow was harvested by flushing the bones with PBS using a 2.5 ml syringe. Any clumps of cells were dispersed with a pipette and passed through 70 μm cell strainer (Fisher Scientific). The cell suspension was centrifuged at 500 × g for 5 minutes, and the pellet was cultured in fresh L929 cell-conditioned DMEM medium for 6 days to differentiate into BMDMs.

### Protein extraction and quantification

Cells washed with PBS were collected into a 1.5 ml Eppendorf tube and thoroughly mixed with lysis buffer (RIPA buffer with 1% protease and phosphatase inhibitor). Cells were incubated at -80 °C for 30 minutes and sonicated for 15 seconds to allow further lysis. Cells were then centrifuged at 15,000 × g for 10 minutes at 4 °C to remove debris and supernatant was collected and stored at -80 °C. PierceTM BCA Protein Assay Kit (Thermo Scientific) was used to quantify proteins as manufacturer's instructions.

### Western blot

Proteins were mixed with 5 × Laemmli buffer and incubated at 100 °C for 10 minutes. Samples were immediately transferred in the ice afterwards. All samples and prestained protein ladder (10-250 kDa, Thermo ScientificTM) were loaded into the columns of NuPAGETM 4-12% Bis-Tris Gel (Invitrogen) placed in the Invitrogen tank containing 1× NuPAGE MOPS SDS running buffer (Novex). The gel was run at 100v for 75 minutes and transferred to a PVDF (Polyvinylidene difluoride) membrane (Millipore) using NuPAGE transfer buffer (Novex) with methanol and antioxidant (Invitrogen) at 35v for 60 minutes. The membrane was blocked with 5% milk-TBST at RT for 1 hour and incubated overnight with TRIB1 (Millipore), and HSP90 (Abcam) diluted in 5% milk-TBST (1:1000 and 1:5000 respectively) at 4 °C. The membrane was then washed with 0.1 v/v TBST for 5 minutes 3 times and incubated with Polyclonal Goat anti-Rabbit Immunoglobulin/HRP, and Polyclonal Rabbit anti-Rat Immunoglobulin/HRP (Dako) diluted in 5% milk-TBST (1:2500 and 1:5000 respectively) at RT for 1 hour. The membrane was then washed with TBST 3 times for 5 minutes, incubated with ECL, and imaged with Bio-Rad imager.

### RNA extraction and quantification

Cells were gently washed twice in PBS and incubated at RT for 5 minutes in 700 μl of QIAzol lysis reagent to homogenate the cells, 140 μl of chloroform was added to cells and RNA extracted using the miRNeasy Mini Kit (Qiagen) according to the manufacturer's instructions. The amount of RNA was quantified using a Nanodrop Spectrophotometer ND1000 and stored at -80 ºC until used for RT-qPCR analysis.

### cDNA synthesis and Real-time quantitative PCR analysis

cDNA was produced with iScript cDNA synthesis kit (Bio-Rad) according to the manufacturer's instructions. Quantitative RT-PCR was performed using primers designed with NCBI BLAST to target human macrophage polarization markers (Supplementary Table 1) and PrecisionPLUS SYBR-Green master mix (Primerdesign). SYBR green master mix with forward and reverse primers were added to each well of a 364-well RT-qPCR plates at a total volume of 5.6 μl, followed by the addition of 5 μl of cDNA (0.4 ng/ul). The plates was then centrifuged for 2 minutes at 2000 rpm and fluorescence measured in a Bio-Rad I-Cycler PCR machine using the protocol provided by the manufacturer. GAPDH and Β-actin were used as housekeeper genes, and the changes in gene expression were obtained using the 2^-ΔΔCT^ method.

### Tissue dissociation

The tumor tissue collected from mice was shredded with scissors, and placed in 5 ml of tumor-dissociation medium (TDM) (IMDM medium, 0.2 mg/ml collagenase IV, 2 mg/ml dispase, 1.25 ug/ml DNase 1) in a 15ml bijou tube, and rotated at 37 °C for 30 minutes. 5 ml of 10% FBS-TDM was added into the tube and passed through a 70 μm filter (Fisher Scientific). The samples were placed directly on ice and centrifuged at 4500 rpm for 5 minutes. The cell pellet was washed three times with PBS and used for flow cytometry analysis.

### Flow cytometry

Total tumor cells were resuspended in PBS and centrifuged at 500 × g for 5 minutes. The samples were resuspended in 100 μl LIVE/DEAD Fixable Blue Dead Cell Stain kit (Invitrogen) and incubated for 15 minutes at RT in the dark, and 200 μl of PBS was added to the tube and centrifuged at 500 × g for 5 minutes. The pellet was resuspended in PBS and centrifuged at 500 × g for 5 minutes. Cells were stained with following antibodies at 1:25 dilution: F4/80 Alexa Fluor 488 (Bio-rad); 1:100 dilutions: CD3 APC (Tonbo Bioscience), MR PE, Ly-6C Alexa Fluor 700, NK1.1 APC-Cy7, Ly-6G Pacific Blue, CD4 PerCP/Cy5.5, CD8 APC-Cy7, CD279 PE (Biolegend); 1:200 dilution: CD274 PE-Cy7 (Biolegend) in FACS buffer (5% FBS in PBS) for 15 minutes at 4 °C in dark. 100 μl FACS buffer was added into the tubes and centrifuged at 500 × g for 5 minutes. Cells were washed with 150 μl FACS buffer and centrifuged at 500 × g for 5 minutes twice. The pellet was resuspended in 200 μl FACS buffer and run with LSRII flow cytometer (Biolegend). Results were analyzed with FlowJo (Treestar).

### Immunofluorescence

The frozen tumor sections were adjusted to RT and then flooded with ice-cold acetone for 10 minutes to fix the tissue. The slides were then air-dried and rehydrated in 0.5% Tween-PBS (PBST) for 3 minutes. The non-specific binding of the secondary antibody was blocked with serum-free protein block (Dako X0909) at RT for 30 minutes and incubated with the following antibodies at 1:25 dilution: F4/80 Alexa Fluor 488 (Bio-rad); 1:50 dilutions: NOS2 (Abcam), CD3 APC (Tonbo Bioscience), CA9 (Abcam), CD68 (Abcam) and TRIB1 (Millipore); 1:100 dilutions: CD31 Alexa Fluor 674 (Biolegend), MR (Abcam), CD4 Alexa Fluor 488 (Biolegend), CD8 PE (Biolegend), IL-15 (Abcam) for 1 hour at RT. The samples were washed twice with PBST for 5 minutes and then incubated with secondary antibody Goat anti-Rabbit IgG (H&L) Dylight 550 (ImmunoReagents), both at 1:50 dilution, Alexa Fluor 488 or 594 goat anti-mouse-IgG or anti-rabbit-IgG secondary antibodies (Invitrogen) at 1:1000 for human TNBC sample, for 1 hour at RT. Slides were washed three times with PBST for 5 minutes and mounted with Antifade mounting medium with DAPI (Life Technology). Slides were kept in the dark overnight at RT and imaged immediately or stored at 4 °C. Random areas of 4-5 images were captured using a Leica AF6000 microscope, or Nikon A1 confocal microscope and cells were manually quantified with ImageJ (NIH).

## Results

### *TRIB1* is highly expressed in tumor-associated macrophages and its expression correlates with response to chemotherapy and patient survival in breast cancer

**Figure 1:**
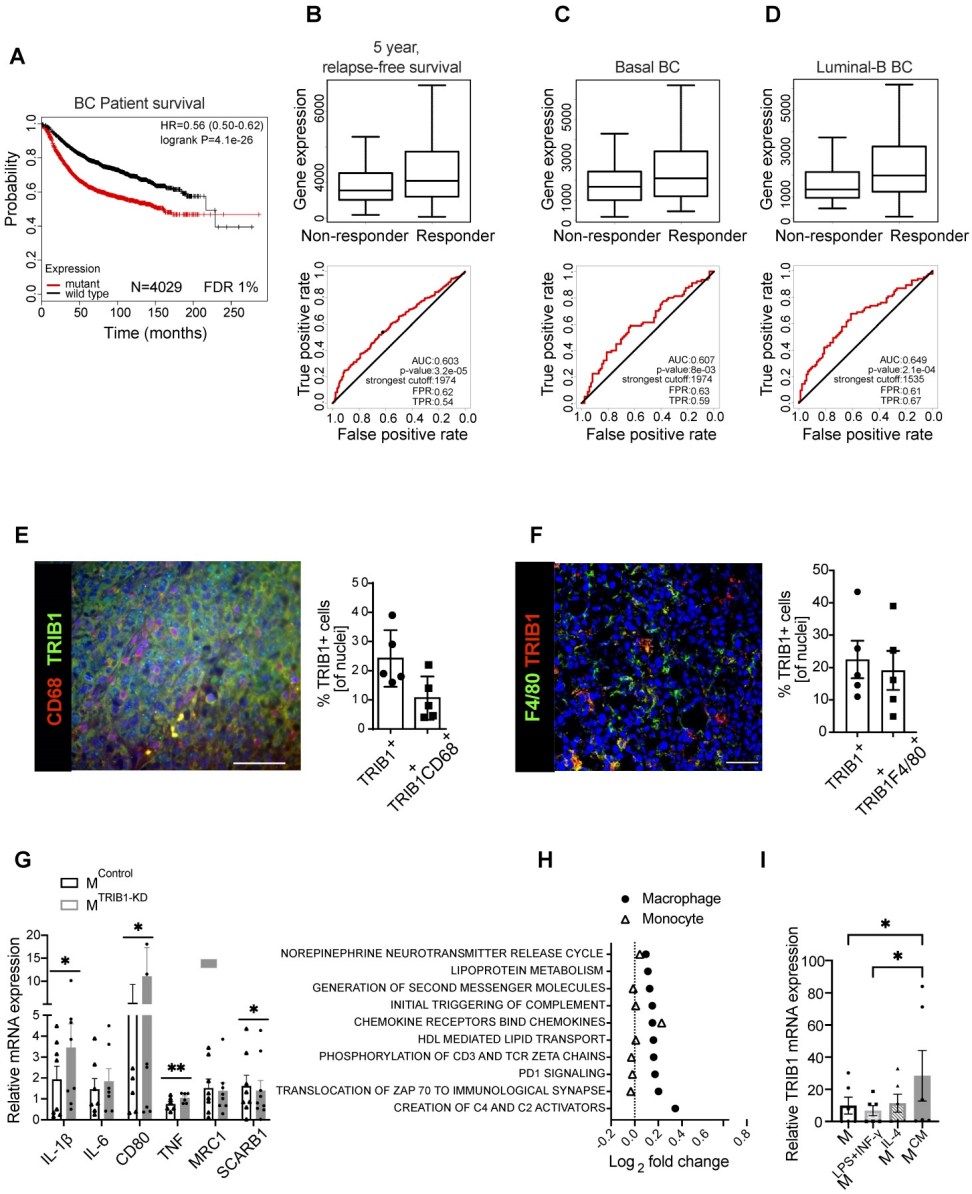
** TRIB1 is associated with overall BC survival and 5-years relapse-free cancer survival after chemotherapy and is a highly expressed protein in Tumor-Associated Macrophages. (A)** TRIB1 mutation-harboring tumors are associated with a poor long-term BC survival. Kapplan-Maier survival plot, Hazard Ratio (HR) and log rank p value are displayed. Red line represents patients harboring the transcriptomic fingerprint of *TRIB1* somatic mutants vs. wild type TRIB represented by a black line. **(B)** *TRIB1* expression in 5-year relapse-free BC survival after anthracycline-based chemotherapy. Note that enhanced TRIB1 expression correlates with a higher 5-year relapse-free BC survival after anthracycline-based chemotherapy. Receiver Operating characteristic (ROC) curves are shown. Area under the curve (AUC), p value, false positive rate (FPR) and true positive rate (TPR). **(B)** All breast cancer patients, **(C)** basal-like and **(D)** luminal-B subtypes. **(E)** Representative image of macrophages in human breast cancer marked with CD68 (red) and TRIB1 (green) (Scale: 100 µm). Quantification of TRIB1 expressing cells and CD68+ cells in the TMA from “E” relative to total cell counts. Data presented as mean±SD. Cells in 5 random fields of views for each tumor were manually quantified using ImageJ.** (F)** Representative image of TRIB1 (red) and F4/80 (green) fluorescence staining of primary murine breast tumors from wild-type (C57/BL6) animals (Scale: 50 µm). Quantification of TRIB1 expressing cells and F4/80+ cells in the TME from “F” relative to total cell counts (n=5 mice/group). Data presented as mean±SEM. Cells in 5 random fields of views for each animal were manually quantified using ImageJ. **(G)** Human MDMs isolated from healthy donors were transfected with either control (M^Control^) or *TRIB1* siRNA for 48 hours (M^TRIB1-KD^). Results of paired t-tests are presented; mean±SEM is plotted; *p < 0.05, **p < 0.01 (n= 6-9 donor/group).** (H)** Pathway analysis of TRIB1 associated genes in monocytes and MDMs in participants of the Cardiogenics Transcriptomic Study. MDM (n= 596) and monocytes (n= 758) were ranked according to *TRIB1* RNA levels and genes that were differentially expressed between the top vs. bottom 25% of the rankings were analyzed with QuSAGE. The 10 most significantly enriched pathways in MDMs are presented. **(I)** *TRIB1* RNA levels of human MDMs unstimulated (M) and stimulated with LPS and INF-γ (M^LPS+INF-γ^), IL-4 (M^IL-4^), and CM (M^CM^) for 24 hours. Results of Friedman test and Dunn's multiple comparison tests are presented; mean±SEM is plotted; *p < 0.05 (n = 6 donors/group).

Whilst *TRIB1* is oncogenic in several cancer settings, its potential importance in BC pathogenesis and response to therapy are largely unknown. To explore the potential role *TRIB1* may play in BC we analyzed the correlation between transcriptomic signatures associated with somatic *TRIB1* mutations and BC survival in a dataset of 6697 patients 29, using the G-2-O algorithm, as previously described 29. This established the association between the prognosis of the specific transcriptomic signature linked with mutations in the protein coding region of *TRIB1* mRNA and patient survival by comparing two cohorts, encoding for wild type or mutant forms of *TRIB1*. The mutant cohort includes only somatic mutations labelled as 'KEEP' by the MutTect judgment algorithm, and present in at least four reads with a minimum of 20-fold read coverage. Mutations are functionally annotated using SNPeff v3.5 30. Both cohorts were compared using a Cox survival analysis, that showed a highly significant reduction in patient survival in tumors with *TRIB1* mutations (Figure 1A, HR 0.56 CI 0.50-0.62; lograk p = 4.1×10^-26^), indicating a worse prognosis for those patients with the transcriptomic signature associated with somatic mutations in the *TRIB1* gene.

Next, we evaluated whether RNA levels of *TRIB1* correlated with relapse-free survival (RFS) in BC patients by interrogating a dataset including 1329 patients with RFS information at 5 years 38. We analyzed different types of therapy, ranging from endocrine treatment (tamoxifen and aromatase inhibitor) to specific anti-HER2 inhibitors (trastuzumab or lapatinib), and several chemotherapy treatments, including taxanes, anthracyclines, Ixabipelone, CMF (cyclophosphamide, methotrexate, fluorouracil), FAC (fluorouracil, adriamycin, citroxan) and FEC (fluorouracil, epirubicin, cyclophosphamide). We found that *TRIB1* expression specifically correlates with 5-year relapse-free survival only in anthracycline-based chemotherapy in BC patients (Figure 1B) and specifically in Basal (Figure 1C) and Luminal-B (Figure 1D) BC subtypes, suggesting it as a putative prognostic marker in this setting. Of note, the luminal-B and basal-like BC subtypes often contain tumors with enhanced proliferation rate and are usually treated with more aggressive chemotherapy combination, including anthracycline-based chemotherapies. Since *TRIB1* is well known for its action to control cell proliferation 39-42, this suggests a potential mechanistic explanation for this association.

Overall, these analyses suggest the involvement of *TRIB1* in response to therapy in breast cancer, and both its loss of function mutations and reduced expression levels render worse prognosis.

Once identified the potential role of *TRIB1* in breast cancer outcome and response to therapy, we aimed to characterize TRIB1 protein expression within mammary tumors, using both specimens from human breast cancer (Figure 1E) and a murine model where BC growth was induced in immune-competent, C57BL/6 mice with an orthotropic injection of a murine, Basal-B BC cell line, E0771 37 (Figure 1F). This analysis revealed that up-to 25% of cells in the tumor expressed high levels of TRIB1 protein and that about 42% (Figure E) of these cells were also positive for the macrophage marker CD68 (Figure 1E). Similarly, 25% of cells in the murine tumor expressed high levels of TRIB1 and 85% (Figure 1F) of these were also positive for the mouse macrophage marker F4/80, together suggesting a potential role for macrophage TRIB1 in regulating tumorigenesis in BC, both in human and murine tumors. Of note, the functional importance of monocyte/macrophages in this mouse model has been demonstrated previously by showing that selective depletion of these cells (but not neutrophils) with gemcitabile led to reduced E0771 tumor growth 43.

In order to gain an initial mechanistic insight into myeloid TRIB1-dependent alterations, relevant to tumor-biology, we used human monocyte-derived macrophages (MDMs) isolated from healthy human blood and transfected them with *TRIB1* siRNA to reduce the expression (M^TRIB1-KD^) to assess the expression of a range of genes known to play an important role in TAM function. Analysis of TRIB1 knockdown efficiency is presented in Figure 1M. This analysis revealed that the knockdown of *TRIB1* in MDMs (for knockdown efficiency of approx. 50%, see Figure 3M) enhances their pro-inflammatory phenotype with a significant increase in levels of expression of *IL-1β* (p < 0.05), *CD80* (p < 0.05), and *TNF* (p < 0.01) mRNA and reduced *SCARB1* expression (p < 0.05) (Figure 1G), in line with changes observed in MDMs stimulated towards an inflammatory (M1) phenotype (M^LPS+INF-γ^, Supplementary Figure 1). These changes are also in line with reported transcriptomic changes in TAMs 44.

Next, we carried out a gene enrichment analysis with QuSAGE 34-36 to identify biological pathways associated with altered *TRIB1* expression in human monocytes (n = 758) and MDMs (n = 596), using data from the Cardiogenics Transcriptomic Study 31-33. Comparison of the 10 most significantly enriched pathways in MDMs (macrophage) and monocytes revealed that most of these pathways were only enriched in macrophages, confirming the distinct regulatory impact of *TRIB1* between these cell types (Figure 1H, Supplementary Table 2). From those pathways significantly associated with *TRIB1* levels in macrophages, such as creation of C4 activators, translocation of ZAP 70 to immunological synapse, PD1 signalling, and phosphorylation of CD3 and zeta chains, have previously all been shown to be involved in the promotion of tumor growth and regulate T-cell activation and polarization 45, 46. In addition, increased PD1 signalling has been reported to increase macrophage proliferation and activation, and inhibit phagocytosis and tumor immunity in TAMs 47, 48.

Finally, treatment of MDMs with cancer cell-conditioned medium (CM) also showed a significant overexpression of *TRIB1* in these cells (M^CM^), compared to control (M^UN^) and M^LPS+INF-γ^ cells (p < 0.05) (Figure 1I), suggesting a potential two-way regulation of *TRIB1* expression between BC cells and tumor macrophages.

### Mammary tumor growth is accelerated by alteration of myeloid* Trib1* levels

**Figure 2:**
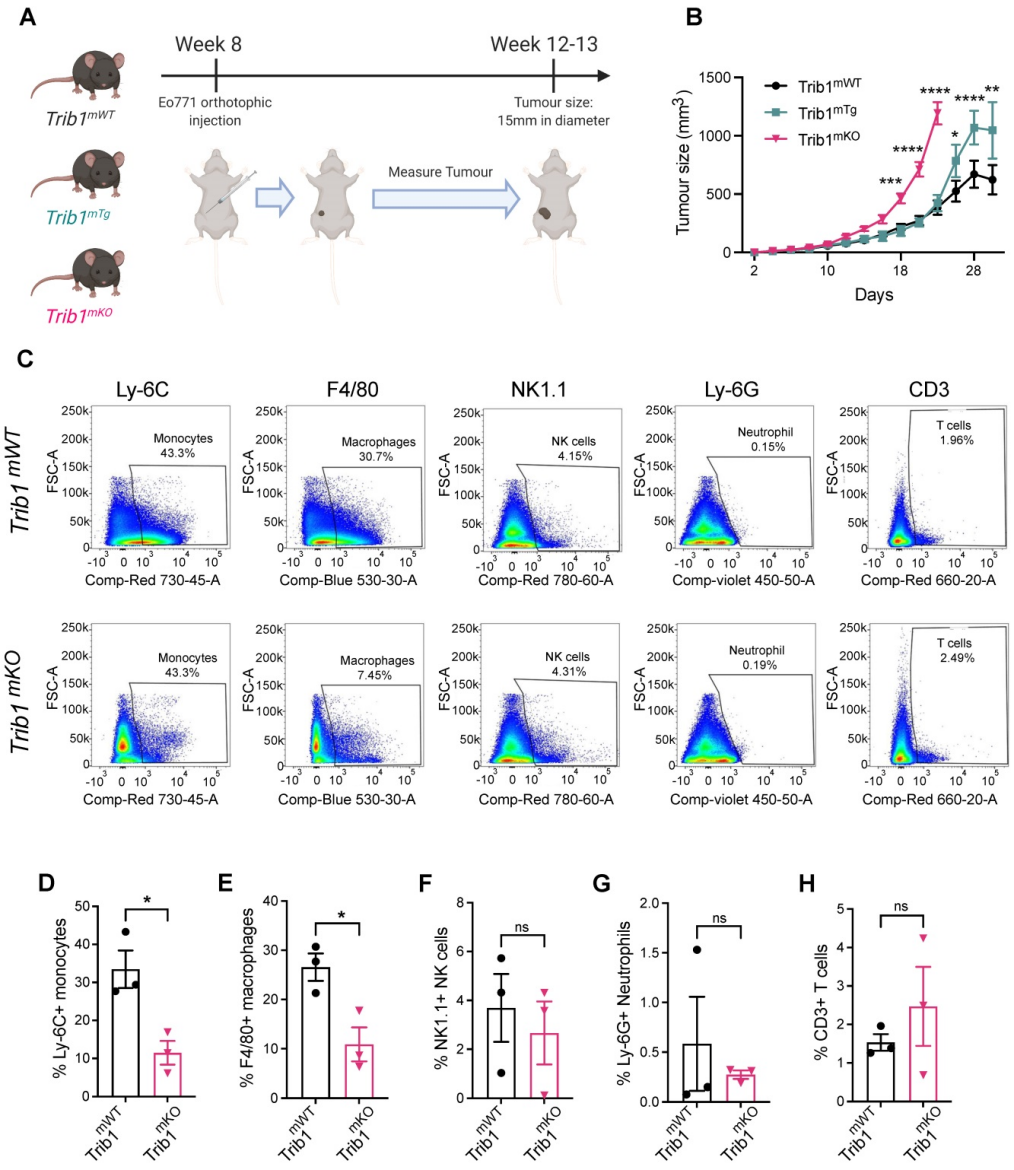
** Reduced myeloid *Trib1* expression accelerates breast tumor growth and inhibits myeloid cell infiltration into the TME. (A)** Development of BC models with altered myeloid *Trib1* expression: Murine BC E0771 cells were injected to the mammary fat pad of myeloid-specific *Trib1* knockout (*Trib1^mKO^*) and transgenic (*Trib1^mTg^*) mice and their respective wild-type (*Trib1^mWT^*) litter-mate controls at the age of 8 weeks. Data was accumulated from > 5 independent experiments, each containing several littermates, including both wild type and m*Trib1*-altered mice. **(B)** m*Trib1*-altered mice developing mammary tumor were routinely monitored and tumor volume was calculated. *Trib1^mTg^* and *Trib1^mKO^* and their litter-mate controls were sacrificed when the tumor reached 15mm in diameter (*Trib1^mKO^* = 22 days; *Trib1^mTg^* = 30 days) and results analyzed by two-way ANOVA; mean±SEM i is plotted; *p < 0.05, **p < 0.01, ***p < 0.001, ****p < 0.0001 (*Trib1^mKO^* n = 5 mice/group; *Trib1^mTg^* n = 12 mice/group, *Trib1^wt^* n = 20 mice/group). **(C)** Post-mortem analysis of immune cell content in *Trib1^mKO^* and respective *Trib1^mWT^* tumors by flow cytometry. Cell markers Ly-6C, F4/80, NK1.1, Ly-6G, CD3 were used to measure the proportion of immune cells **(D-H)** Quantification of immune cell content in *Trib1^mKO^* and respective *Trib1^mWT^* tumors. Results of Welch's t-test are presented; mean±SEM is plotted; *p < 0.05 (n = 3 mice/group).

Based on the above evidence of potential association between *TRIB1* and BC, we hypothesized that myeloid TRIB1 expression may influence the aggressiveness of BC, and thus modulation of *Trib1* expression in these cells would alter tumor growth. The complex role of tumor resident macrophages has been studied extensively, and has recently been proposed that anthracycline-based chemotherapy may lead to an effective anti-tumor immunity via macrophage-mediated effects 49. Our observations presented above may link high TRIB1 levels mechanistically to the enhanced responses to anthracycline-based chemotherapy, prompting us to characterize how altered Trib1 expression in myeloid cells may alter BC tumor growth. To test this, we used myeloid-specific *Trib1* overexpressing (*Trib1^mTg^*) and knockout (*Trib1^mKO^*) mice and*.* their littermate controls, on an immune-competent, C57BL/6 background. We have recently reported details of the development and initial characterization of these mouse lines 19. We modelled BC growth, using these myeloid-specific mouse lines and a murine breast cancer cell line, E0771, that has recently been reported to be a luminal-B subtype 37, thus closely resembling the human BC subtype, where *TRIB1* expression levels are correlated with response to chemotherapy (Figure 1D). Mammary fat pads of 8-week-old mice were injected with E0771 cells, and the rate of tumor growth measured (Figure 2A). Interestingly, whilst *Trib1^mKO^* tumor growth rate accelerated from an early stage and significantly increased from day 18 (p < 0.001), reaching 15 mm in diameter (1770 mm^3^ volume) at day 22, *Trib1^mTg^* animals displayed a tumor growth rate similar to wild-type littermates until day 24 (Figure 2B). At this point, the rate of tumor volume growth became slower in wild-type animals, compared to *Trib1^mTg^* animals. In the *Trib1^mTg^* group, tumor continued to grow and reached 15 mm in diameter at day 30 (p < 0.01) (Figure 2B). Tumors from both cohorts were collected when they reached 15 mm in diameter for further analysis, together with corresponding WT littermate controls.

### Myeloid*-Trib1* knockout reduces macrophage infiltration and promotes oncogenic cytokine expression in TAMs

Tumors from *Trib1^mKO^* animals were initially analyzed by flow cytometry to investigate populations of immune cells in the tumor microenvironment (TME) (Figure 2C, Supplementary Figures 2A and C). This analysis revealed that tumors developed in *Trib1^mKO^* animals had a significantly reduced infiltration of both Ly-6C^+^ monocytes and F4/80^+^ macrophages into the TME (p < 0.05) (Figure 2D-E). In contrast, the percentage of Ly-6G^+^ neutrophils, NK1.1^+^ NK cells, and CD3^+^ T-cells and its subtypes (CD4^+^ naïve and CD8^+^ cytotoxic T cells) in the tumor were not altered between *Trib1^mWT^* and *Trib1^mKO^* (Figure 2F-H, Supplementary Figure 2B and D).

**Figure 3:**
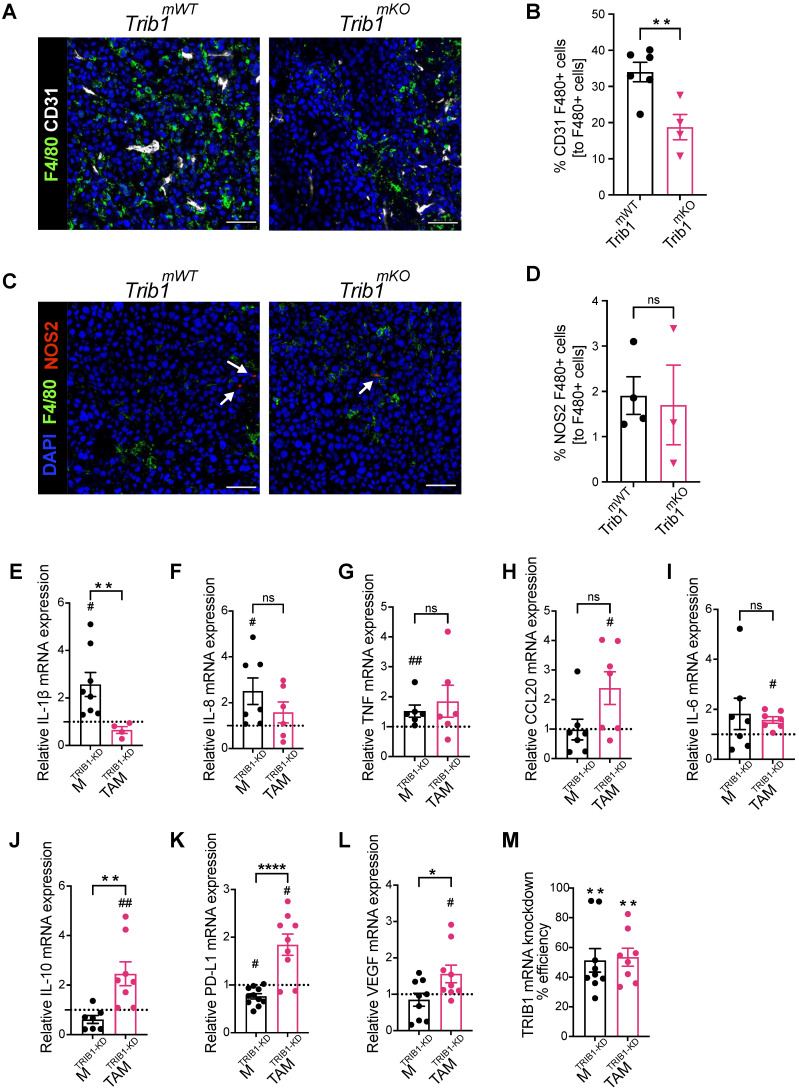
** Myeloid *Trib1* knockout inhibits perivascular TAM infiltration, and *TRIB1* knockdown in TAMs enhances oncogenic cytokine expression. (A)** Representative images of CD31 (white) and F4/80 (green) fluorescence staining in *Trib1^mKO^* and respective *Trib1^mWT^* tumors (Scale: 50 µm); **(B)** Quantification of perivascular TAMs (F4/80+ CD31+) in *Trib1^mKO^* tumors relative to the total number of F4/80+ TAMs. Cell numbers were quantified manually from 5 randomly taken field of views using ImageJ. Results of unpaired t-test are presented; mean±SEM is plotted; **p < 0.01 (n = 4-6 mice/group); **(C)** NOS2 (red) and F4/80 (green) fluorescence staining in *Trib1^mKO^* and respective *Trib1^mWT^* tumors (Scale: 50 µm). NOS2 (red) staining is marked with white arrows. **(D)** Quantification of pro-inflammatory TAMs (F4/80+ NOS2+) in *Trib1^mKO^* tumors relative to the total number of F4/80+ TAMs. Cell numbers were quantified manually from 5 randomly taken field of views using ImageJ. Results of unpaired t-test are presented; mean±SEM is plotted (n = 3-4 mice/group). **(E-M)** Human MDMs isolated and differentiated from blood were transfected with either non-targeting control or *TRIB1* siRNA for 48 hours and either left unpolarized or polarized to TAMs using the CM for 24 hours. (**E-L**) Expression values of *TRIB1* knockdown M and TAMs were initially compared to their controls (shown as fold difference to the dotted line), followed by analysis of difference between M^TRIB1-KD^ and TAM^TRIB1-KD^. (**M**) The efficiency of *TRIB1* siRNA transfection 48 hours after *TRIB1* siRNA transfection (M^TRIB1-KD^) and TAM polarization (TAM^TRIB1-KD^) were assessed. Results of paired and unpaired t-tests are presented; mean±SEM is plotted; *p < 0.05, **p < 0.01, ****p < 0.0001 represent p-values between M^TRIB1-KD^ *vs*. TAM^TRIB1-KD^ whilst #p < 0.05 ##p < 0.005 represent p-values of M^TRIB1-KD^ and TAM^TRIB1-KD^ to their respective control (M^Control^ and TAM^Control^) (n = 4-9 donor/group).

In order to explore the potential mechanisms of the observed accelerated mammary tumor growth and its links with the reduced monocyte and macrophage infiltration in *Trib1^mKO^* mice, we further assessed the localization of macrophages and their phenotype using immuno-fluorescence staining and flow cytometry (Figure 3A and 3C, and Supplementary Figure 2E). Perivascular TAMs (PV TAMs) are in close contact with blood vessels (within 250 μm radius) and play a crucial role in angiogenesis in mammary cancers as well as metastasis and intravasation of cancer cells 50, 51. Staining of TAMs and endothelial cells for F4/80 and CD31, respectively, revealed a significant reduction of PV TAMs in *Trib1^mKO^* tumors (Figure 3B). However, although an increase in pro-inflammatory macrophages has been reported previously both in full-body and myeloid-specific *Trib1* knockout animals 19, 21, inhibition of myeloid *Trib1* expression did not alter the ratio of NOS2^+^ pro-inflammatory TAMs and mannose receptor (MR)^+^, anti-inflammatory TAMs in the TME (Figure 3D, Supplementary Figure 2F, Supplementary figure 3), suggesting that the reduced TAM numbers, rather than their altered inflammatory phenotypes, may have contributed to the accelerated tumor growth.

In order to gain a mechanistic insight into how *TRIB1* regulates monocyte-derived macrophages and the impact of reduced *TRIB1* expression on re-educating macrophages towards TAMs, human MDMs were transfected with siRNA against *TRIB1* (M^TRIB1-KD^), followed by a treatment with tumor-conditioned medium (CM) (TAM^TRIB1-KD^) which resulted in ~50% reduction in TRIB1 expression (Figure 3M). Expression of key cytokines were assessed by RT-qPCR, revealing that expression of pro-inflammatory cytokines *IL-1β* (p < 0.05)*, IL-8* (p < 0.05)*,* and *TNF* (p < 0.01) were significantly increased in M^TRIB1-KD^ but were not altered in TAM^TRIB1-KD^, compared to non-targeting siRNA transfected MDMs (Figure 3E-G, Supplementary Figure 4A-C and I-K). This observation is in line with data presented in Figure 3C-D, thus suggesting that macrophage TRIB1-deficiency does not alter the inflammatory properties of TAMs *per se*. Of interest, *TRIB1* knockdown in MDMs (M^TRIB1-KD^) enhanced expression of IL1β and TNF mRNA (Figure 1G), emphasizing that cancer cell secreted factors that were used to re-educate M^TRIB1-KD^ to TAM^TRIB1-KD^ are effectively altering the phenotypes of these cells. In contrast, *TRIB1* knockdown in TAM^TRIB1-KD^ significantly induced expression of several oncogenic cytokines, including *CCL20* (p < 0.05)*, IL-6* (p < 0.05)*, IL-10* (p < 0.01)*, PD-L1* (p < 0.05), and *VEGF* (p < 0.05), compared to M^TRIB1-KD^ (Figure 3H-L, Supplementary Figure 4D-H and L-P). Notably, *IL-10* (p < 0.01)*, PD-L1* (p < 0.0001) and *VEGF* (p < 0.05) expression were significantly increased in TAM^TRIB1-KD^ compared to M^TRIB1-KD^ (Figure 3J-L), suggesting the myeloid *TRIB1* is an important regulator of oncogenic cytokine expression in TAMs, downstream of signals secreted by tumor cells.

### Overexpression of *Trib1* reduces hypoxic TAM infiltration and inhibits pro-inflammatory TAM polarization

**Figure 4:**
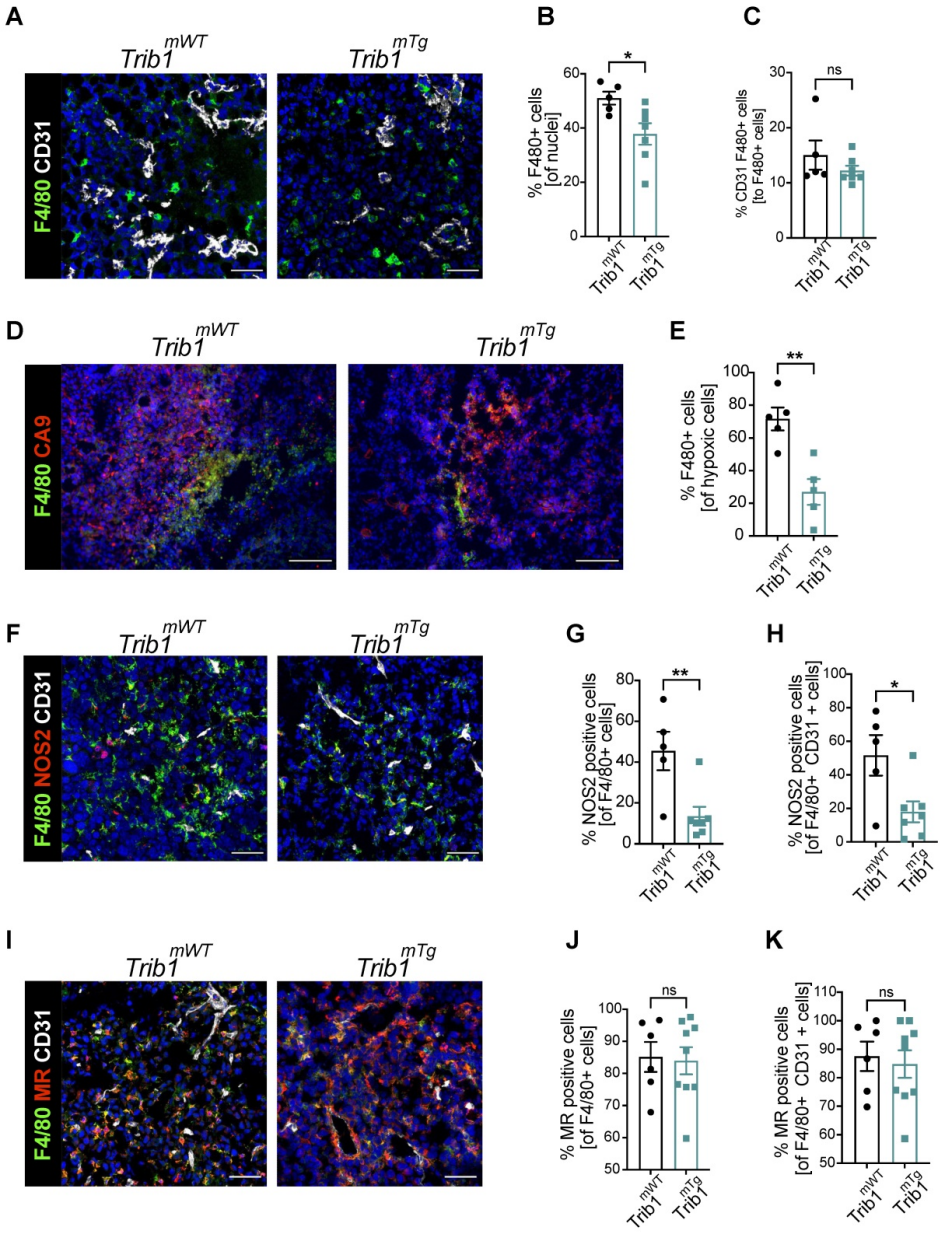
** Overexpression of myeloid *Trib1* reduces hypoxic TAM numbers in the TME and inhibits TAM polarization towards a pro-inflammatory phenotype.** Post-mortem analysis of TAMs and their subtypes based on the location and phenotypes using immunofluorescence staining. **(A)** Representative images of perivascular TAM CD31 (white) and F4/80 (green) (Scale: 50 µm), **(D)** hypoxic TAM CA9 (red) and F4/80 (green) (Scale: 100 µm), **(F)** pro-inflammatory TAM CD31 (white), NOS2 (red), and F4/80 (green) (Scale: 50 µm), and **(I)** anti-inflammatory TAM CD31 (white), MR (red), and F4/80 (green) (Scale: 50 µm) in *Trib1^mWT^* and *Trib1^mTg^* tumors. Cells were quantified manually from 4-5 randomly taken fields of view using ImageJ. Percentage of TAMs and TAMs classified based on their location (vessels and hypoxia) were Trib1 overexpression inhibited TAMs, both perivascular and hypoxic TAMs, pro-inflammatory TAMs and pro-inflammatory TAMs in the vessels in tumors compared to WT **(B, C, E, G, H respectively)**. Percentage of anti-inflammatory TAMs did not alter in *Trib1^mTg^* **(J, K)**. Results of unpaired t-test are presented; mean±SEM is plotted; *p < 0.05 **p < 0.01 (n = 5-9 mice/group).

The above analysis of tumor growth in *Trib1^mTg^* mice (Figure 2A) revealed that elevated myeloid-*Trib1* levels lead to an increase in tumor size at advanced stages of tumor growth. To gain a mechanistic understanding of this effect, TAM localization and phenotypes in *Trib1^mTg^* TME were investigated using immune-fluorescence staining. Carbonic anhydrase IX (CA9) is a cell-surface glycoprotein in the tumor, expression of which is induced by hypoxia and has been shown to be involved in cancer progression 52. Thus, staining for CD31 and CA9 were used, together with F4/80 to identify PV TAMs* vs*. TAMs residing in hypoxic areas (Figure 4A-E, and Supplementary Figure 5). Pro- and anti-inflammatory markers (NOS2 and MR, respectively) were used to characterize TAM phenotypes in *Trib1^mTg^* tumors (Figure 4F-K). Similar to *Trib1^mKO^*, we observed a significant overall reduction of F4/80+ TAM numbers in *Trib1^mTg^* (p < 0.05) (Figure 4B). Whilst there was no difference in CD31^+^ F4/80^+^ PV TAMs (Figure 4C), a significant reduction was observed in CA9^+^ F4/80^+^ hypoxic TAMs in *Trib1^mTg^* tumors, compared to *Trib1^mWT^* (p < 0.005) (Figure 4E). Further, staining of TAMs with phenotypic markers (NOS2 and MR) also demonstrated a reduction in NOS2^+^ TAM numbers (p < 0.005), including PV TAMs (p < 0.05) (Figure 4F-H) but did not alter the ratio of MR^+^ TAMs (Figure 4I-K).

### *Trib1^mTg^* tumors display reduced T cell infiltration and reduced IL-15 expression

**Figure 5:**
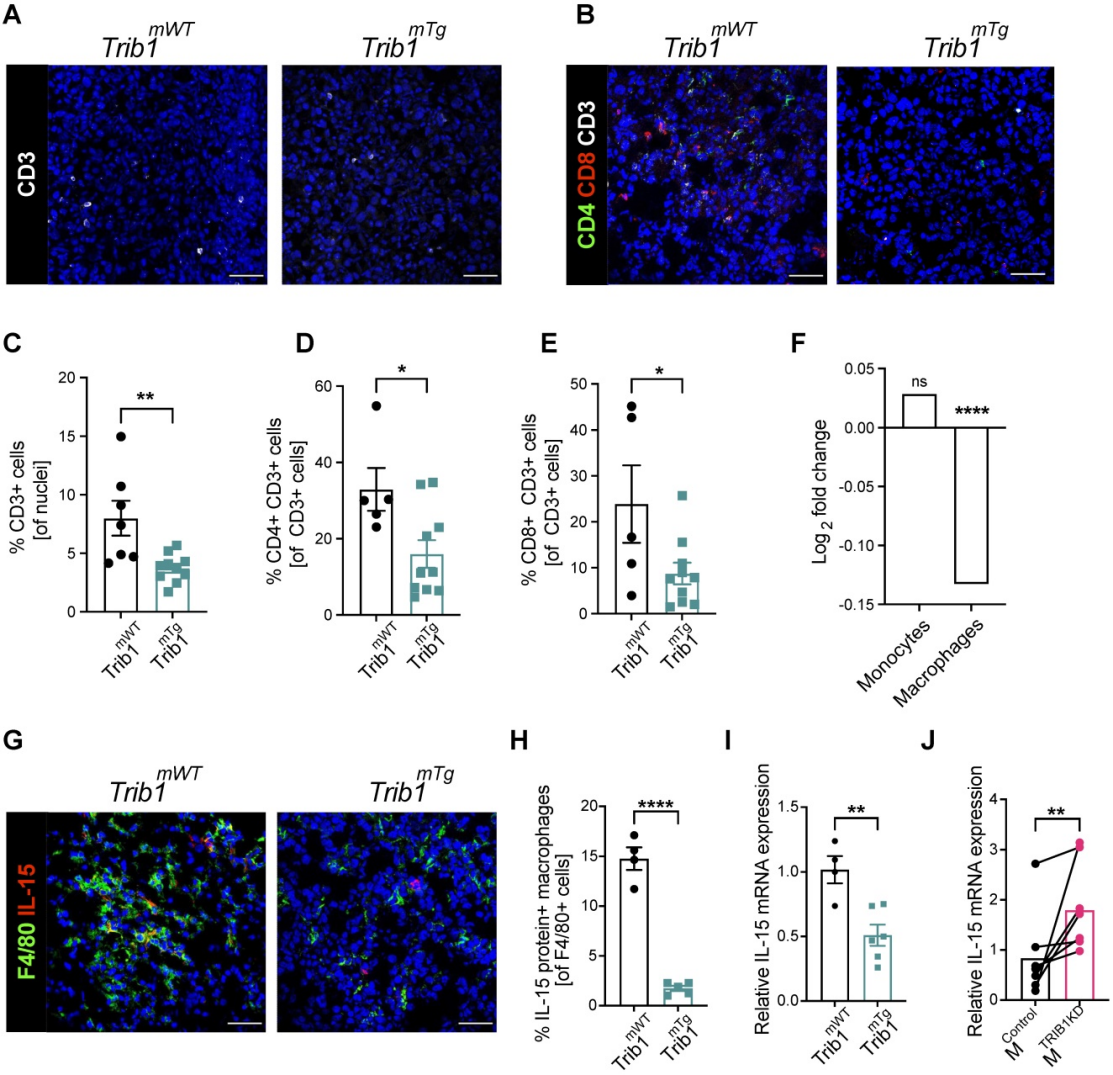
** Myeloid *Trib1* overexpression impairs IL-15 expression and significantly reduces T-cells in the TME. (A-B)** Representative images of CD3 (white) staining in *Trib1^mTg^* and respective *Trib1^mWT^* tumors (Scale: 50 µm); and CD3 (white), CD4 (green), and CD8 (red) fluorescence staining in *Trib1^mTg^* and respective *Trib1^mWT^* tumors (Scale: 50 µm). Cells were quantified manually from 4-5 randomly taken field of views using ImageJ. **(C)** Quantification of T-cells (n = 7-10 mice/group), and** (D, E)** CD4+ *naïve* and CD8+ cytotoxic T-cells (n = 5-11 mice/group) in *Trib1^mTg^* and respective *Trib1^mWT^* tumors. Results of unpaired t-test is presented; mean±SEM is plotted; *p < 0.05, **p < 0.01. **(F)** *IL-15* expression in human monocytes and MDMs from participants of the Cardiogenics Transcriptomic Study. MDM (n= 596) and monocytes (n = 758) were ranked according to *TRIB1* RNA levels and *IL-15* expressed between the top vs. bottom 25% of the samples were plotted. Results of FDR adjusted p-values are presented as ****p < 0.0001. **(G)** Representative images of IL-15 (red) and F4/80 (green) fluorescence staining in *Trib1^mTg^* and respective *Trib1^mWT^* tumors (Scale: 50 µm). Cells were quantified manually from 4 randomly taken field of views using ImageJ. **(H)** Quantification of TAMs expressing IL-15 in *Trib1^mTg^* and respective *Trib1^mWT^* TME relative to the total number of TAMs. Results of unpaired t-test is presented; mean±SEM is plotted; ****p < 0.0001 (n = 4-5 mice/group). **(I)** Mouse BMDMs isolated from *Trib1^mWT^* and *Trib1^mTg^* animals were analyzed with RT-qPCR. RNA level of *Il-15* in mouse BMDMs. Results of unpaired t-test is presented; mean±SEM is plotted; **p < 0.01 (n = 4-6 mice/group). **(J)** Human MDMs isolated and differentiated from blood were transfected with either control or *TRIB1* siRNA for 48 hours and *IL-15* RNA expression was analyzed. Results of paired t-test is presented; mean is plotted; **p < 0.01 (n= 6 donor/group).

Recruitment of T-cells to the TME is a central mechanism for inhibition of tumorigenesis and cytokines secreted by TAMs play a key role in this process 53, 54. Our above data demonstrates that reduced *vs.* elevated expression of *mTrib1* leads to distinct changes in TAM phenotypes and have also shown that recruitment of T-cells in *Trib1^mKO^* tumors is unaltered (Figure 2H and Supplementary Figure 2A-D). Thus, we next tested whether the observed alterations in TAM numbers and phenotypes affect T-cell recruitment in *Trib1^mTg^* animals. Fluorescence staining was used to identify changes in CD3^+^ T-cell numbers and populations of CD4^+^ *naïve* and CD8^+^ cytotoxic T-cells (Figure 5A and B, Supplementary Figure 6), revealing a significant reduction in the overall number of CD3^+^ T-cells in the *Trib1^mTg^* TME, compared to *Trib1^WT^* (p < 0.01) (Figure 5C). Furthermore, the proportion of both CD4^+^ CD3^+^ *naïve* T-cells and CD8^+^ CD3^+^ cytotoxic T-cells were significantly reduced in *Trib1^mTg^* (p < 0.05) (Figure 5D-E).

In order to identify *mTrib1*-dependent mechanisms that may explain an impaired T-cell recruitment to the tumor, we have assessed the correlation between *TRIB1* levels and genes that have been shown to regulate T-cell recruitment in the Cardiogenics Transcriptomic Study 31-33. This analysis of approx. 600 independent samples revealed that high *TRIB1* levels correlate very significantly with a reduced IL-15 expression in human macrophages (but not in monocytes) (Figure 5F). IL-15 is a cytokine expressed by myeloid cells crucial for the development, function and survival of T-cells. IL-15 stimulates tumor-specific T-cell responses, increases cellular growth, inhibits apoptosis, and enhances immune cell activation, and as a consequence, promotes anti-tumor responses 55. Recent work reported by Pavlakis *et al.* demonstrated that peritumoral delivery of heteromeric IL-15 led to an effective suppression of E0771 orthopically implanted tumors 56, 57, thus, demonstrating the direct anti-tumorigenic properties of IL-15 in this *in vivo* model of BC. Therefore, we next assessed IL-15 in *Trib1^mTg^* TAMs by fluorescence staining (Figure 5G, Supplementary Figure 6). Quantification of IL-15^+^ TAMs revealed that overexpression of *Trib1* led to significantly reduced IL-15 expressing TAM numbers in the TME (p < 0.01) (Figure 5H). To substantiate that *TRIB1* is a direct regulator of *IL-15* expression in myeloid cells, RT-qPCR analysis was performed in BMDMs isolated from *Trib1^mTg^* showing that enhanced *Trib1* expression led to a significant reduction in *IL-15* expression (Figure 5I). In line with this, a transient knockdown of *TRIB1* with siRNA transfection in human MDMs from healthy human participants significantly increased *IL-15* expression (p < 0.01) (Figure 5J).

## Discussion

It is now widely recognized that TAMs are a crucial component of TME and the number of these cells is associated with cancer cell resistance to therapy, poor patient survival and prognosis 6-9. However, the molecular mechanisms that shape TAM phenotype, and thus determine whether they are pro-tumorigenic or promoting anti-tumor immune responses, are poorly understood.

The pseudokinase protein, TRIB1, has been reported as a potential regulator of macrophage phenotypes. It is highly expressed in the myeloid lineage and is associated with altered tissue macrophage phenotypes 19, 21, 58. Although the effect of *TRIB1* in TAMs has not been elucidated, previous studies investigated *TRIB1* as an oncogene in several contexts 24-26, including prostate and colon cancer and also associated it with sensitivity of breast cancer cells to TNF-related apoptosis-inducing ligand (TRAIL) -induced apoptosis 27, 59, 60.

Putting together these published data, our observations that *TRIB1* expression is associated with patient survival and therapy responses in breast cancer patients and that the majority of TAMs highly express TRIB1 in a murine model of BC, as well as in human BC specimens, we hypothesized that altered TRIB1 expression in myeloid cells may modulate TAM phenotypes. As a consequence, *mTRIB1* would mechanistically contribute to breast cancer tumor growth, as well as to response to chemotherapy.

Of note, the TRIB1-dependent prediction of response to therapy is only observed in those tumors with a higher rate of proliferation, such as the basal-like and luminal B phenotypes. The latter is characterized by the dual expression of the estrogen and HER2 receptor that constitutes two druggable oncogenic vulnerabilities 61. Chemotherapy, and particularly anthracyclines, is a backbone treatment in this disease and it's known that the immunologic state can modulate the efficacy to these agents through the presence of different immune populations and secreted factors 62.

We used E0771 cells in this study, that have been shown to express estrogen receptor (ER), progesterone receptor (PR) and ERBB2 and are therefore classified as a luminal B subtype> This subtype is found in 30-40% of BCs and generally known to be more aggressive than luminal A BCs 37. Of note, our analysis of patient survival showed that *TRIB1* expression is elevated in tumors responding to chemotherapy in Luminal B BC, compared to non-responders, further justifying the choice of this murine model.

TAM phenotype in the solid tumor is critical for tumor growth, where proteins secreted by cancer cells (such as IL-4, IL-10, and CSF-1) drive TAMs towards an anti-inflammatory phenotype that promotes angiogenesis and immunosuppression 11, 63. However, pro-inflammatory TAMs can also play oncogenic roles, particularly at the early phases of tumor growth, linked to hypoxia. Abundant infiltration of pro-inflammatory TAMs has been observed in early tumor development, and the expression of TNF and activation of PGC-1α and AMPK was shown to promote glycolysis and exacerbate tumor hypoxia 54, 64. The importance of hypoxic signals has also been evidenced where knockout of HIF-1α reduced the proliferation of BC cells *in vitro* as well as primary breast tumor volume by 60% *in vivo* 65. Hypoxic TAMs have also been shown to secrete angiogenic proteins, with HIF-1α stimulating pro-angiogenic functions in TAMs, thus facilitating tumor vascularization 17, 63. Perivascular (PV) TAMs express high levels of MRC1 and VEGF to facilitate tumor angiogenesis, and help formation of paracrine feedback loops (CSF1 from cancer cells, EGF from TAMs, and HGF from endothelial cells) to initiate metastasis and intravasation of cancer cells at the TME of metastasis 50, 51, 66, 67. Thereby, although *Trib1^mKO^* and *Trib1^mTg^* both demonstrated a significant reduction in TAM infiltration overall in our BC models, detailed analysis of *Trib1^mTg^* revealed a significant and localized reduction of TAM numbers in hypoxic areas, as well as inhibition of pro-inflammatory TAM polarization in the TME, both of which mechanisms that may contribute to the observed late acceleration of mammary tumor growth.

In contrast, *Trib1^mKO^* reduced infiltration of PV TAMs, but did not alter the number of NOS2 positive macrophages in the tumor. Instead, *in vitro TRIB1* knockdown in a model of human TAMs revealed that inhibition of *TRIB1* enhances expression of oncogenic cytokines in TAMs, which are involved both in cancer cell survival and immune suppression. Increased IL-6 expression in TAMs was reported to promote cancer cell survival resistance to hypoxia 68; IL-10 is known to suppress immune surveillance, inhibit apoptosis, and to enhance migration of cancer cells 69, 70; overexpression of PD-L1 disrupts T-cell proliferation and function 71 and VEGF enhances BC growth and angiogenesis 72. These observations are in line with our previous work, where we have shown that myeloid *Trib1*-deficiency alters macrophage function (in that case, formation of foam cells in the atherosclerotic plaque), rather than a clear shift in inflammatory status of *Trib1^mKO^* cells 19.

TAMs interact with T-cells in TME to suppress T cell-driven cytotoxic immune response and promote tumor growth. Previous studies reported that TAMs impair CD8^+^ T-cell activation and proliferation, and depletion of TAMs in the TME enhances the infiltration of both *naïve* and cytotoxic T-cells 54, 73. Tumor-infiltrating T-cells enter tumor at an early stage as *naïve* CD4^+^ T-cells, followed by macrophage infiltration and contribute to early tumor rejection and/or anti-tumor effects through promoting senescence and tumor apoptosis via secretion of cytokines (such as IFN-γ and TNF) and interact with macrophages, NK cells and CD8^+^ T-cells to enhance tumor eradication 53. Interestingly, a recent study from Carrero *et al.* have shown that most myeloid cells in the tumor TME express high levels of IL-15 74, proposing that these stromal cells may be a critical source of this anti-tumor cytokine. In this study, we identified a significantly reduced infiltration of both CD4^+^ *naïve* and CD8^+^ cytotoxic T-cells into the TME in *Trib1^mTg^* animals, despite TAM infiltration being inhibited. Our mechanistic analysis revealed that regulation of T cell infiltration may be due to a previously unrecognized role of myeloid-TRIB1 as a critical regulator of IL-15 expression.

We have reported previously that *Trib1^mTg^* mice also express the transgene in neutrophils and Lyz2-Cre expression is also expected to delete the *Trib1* allele in the *mKO* animals in these cells, in addition to monocyte/macrophages 19. However, we focused our analysis reported here on monocyte/macrophages only, for a number of reasons. First, we have shown that *Trib1^mT^* and *Trib1^mKO^* animals have unaltered neutrophil numbers 19. Second, relevant to our specific *in vivo* model of BC, as well as the *TRIB1*- associated patient survival, most Luminal primary BC tumors in patients have been shown not to contain tumor-associated neutrophils (TAN) 75. Whilst a number of analyses have investigated the prognostic value of neutrophil-to-lymphocyte ratio (NLR), these (mostly retrospective) reports yielded conflicting data 76, 77 and prospective studies concluded that NLR has no prognostic value in most BC subsets after correcting for clinic-pathological factors 78. More recently, a transcriptomic analysis of BC datasets has shown that the proportion of neutrophils was significantly higher in BC cases with a higher grade and of the luminal B, TNBC and HER2+ subtypes but was not associated with tumor size or axillary lymph node metastasis 79.

**Figure 6:**
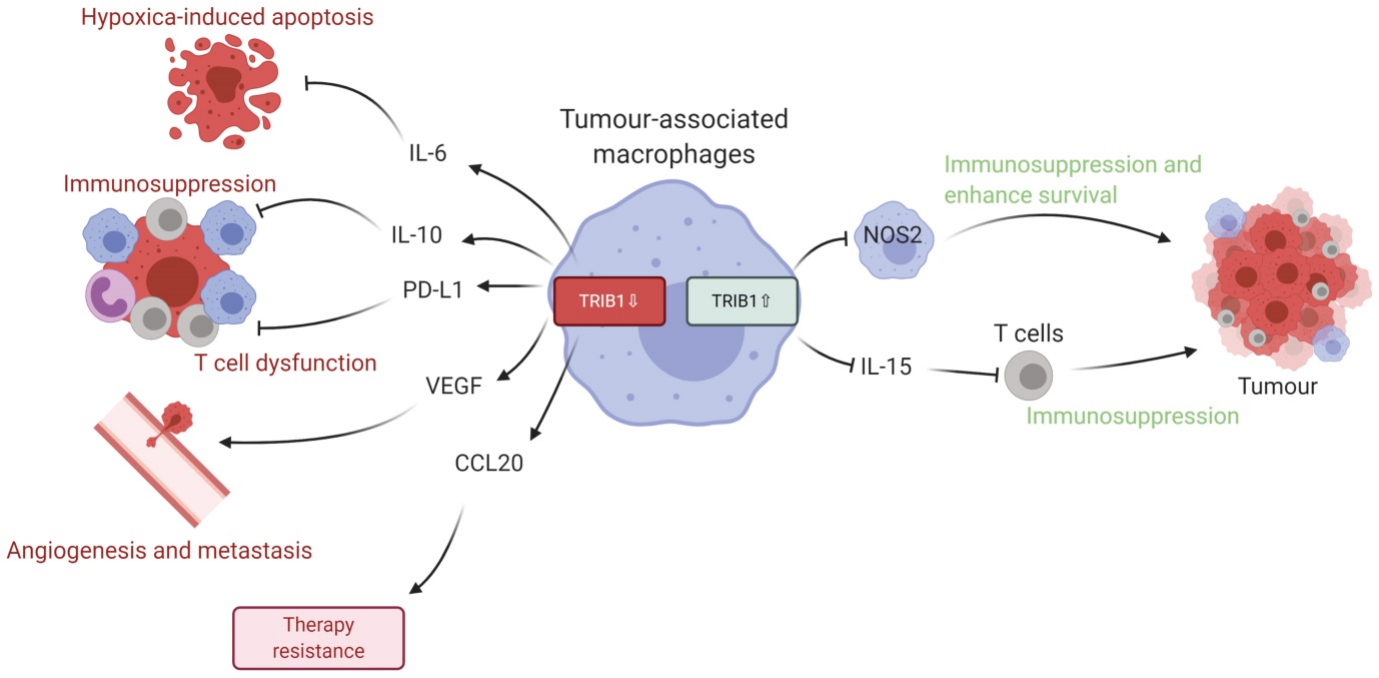
** Schematic representation of TRIB1 mediated TAM regulation.** Alteration of TRIB1 distinctly regulates TAMs to enhance tumor growth. Reduction of TRIB1 accelerates oncogenic cytokine IL-6, IL-10, CCL20, PD-L1, and VEGF expressions in TAMs which take part in the inhibition of cell apoptosis, immune cell dysfunction, angiogenesis, and develop resistance to therapies. Whilst the overexpression inhibits pro-inflammatory TAM in the TME to suppress the immune response and enhance cancer cell survival. TRIB1 conversely influence IL-15 expression, which reduces T cell infiltration and potentially disrupts T cell-induced immune responses.

Given the data we present in this study, we propose that dysregulated levels of *TRIB1* in myeloid cells leads to accelerated tumor growth via distinct molecular mechanisms (Figure 6). Specifically, *TRIB1* expression is associated with lower levels of anti-tumorigenic factors such as *IL6* (that promotes hypoxia-induced apoptosis), *IL10* and *PD-L1* (that regulate T cell immunosuppression), *CCL20* (that influences response to chemotherapy) and proangiogenic *VEGF*. Conversely, higher levels of *TRIB1* are linked to increased tumor cell survival (via *NOS2*) and decreased T cell-mediated immunosuppression (via *IL15*). More generally, this study exemplifies how alterations in the expression of the same gene in TAMs may have opposing consequences at different stages of tumor development. Whilst it is to be formally tested in future studies, we speculate that *TRIB1* expression changes in TAMs could be associated with the initiation and/or with the growth of the tumor and adaptation to lack of nutrients, as well as to hypoxic environment. Nevertheless, knockout of myeloid-*TRIB1* upregulates the expression of oncogenic cytokines in TAMs whilst its overexpression modifies TAM phenotype and T-cell composition in the TME, both enhancing tumor growth. Such data reinforce the general concept for the complex role of TAMs in BC and analysis of consequences for altered *TRIB1* expression highlight potential diagnostic/prognostic markers and therapeutic markers for anti-cancer immunotherapy. In addition, our findings also support the idea that enhanced *TRIB1* expression could be explored as a potential biomarker in BC that might help to predict response chemotherapy.

## Supplementary Material

Supplementary figures and tables.
